# Experimental Characterization of the FRCM-Concrete Interface Bond Behavior Assisted by Digital Image Correlation

**DOI:** 10.3390/s21041154

**Published:** 2021-02-06

**Authors:** Dario De Domenico, Antonino Quattrocchi, Damiano Alizzio, Roberto Montanini, Santi Urso, Giuseppe Ricciardi, Antonino Recupero

**Affiliations:** Department of Engineering, University of Messina, Vill. S. Agata, 98166 Messina, Italy; aquattrocchi@unime.it (A.Q.); dalizzio@unime.it (D.A.); roberto.montanini@unime.it (R.M.); surso@unime.it (S.U.); gricciardi@unime.it (G.R.); arecupero@unime.it (A.R.)

**Keywords:** Digital Image Correlation (DIC), fabric reinforced cementitious mortar (FRCM) system, bond behavior, notched beams, failure mode, strain distribution

## Abstract

Digital Image Correlation (DIC) provides measurements without disturbing the specimen, which is a major advantage over contact methods. Additionally, DIC techniques provide full-field maps of response quantities like strains and displacements, unlike traditional methods that are limited to a local investigation. In this work, an experimental application of DIC is presented to investigate a problem of relevant interest in the civil engineering field, namely the interface behavior between externally bonded fabric reinforced cementitious mortar (FRCM) sheets and concrete substrate. This represents a widespread strengthening technique of existing reinforced concrete structures, but its effectiveness is strongly related to the bond behavior between composite fabric and underlying concrete. To investigate this phenomenon, a set of notched concrete beams are realized, reinforced with FRCM sheets on the bottom face, subsequently cured in different environmental conditions (humidity and temperature) and finally tested up to failure under three-point bending. Mechanical tests are carried out vis-à-vis DIC measurements using two distinct cameras simultaneously, one focused on the concrete front face and another focused on the FRCM-concrete interface. This experimental setup makes it possible to interpret the mechanical behavior and failure mode of the specimens not only from a traditional macroscopic viewpoint but also under a local perspective concerning the evolution of the strain distribution at the FRCM-concrete interface obtained by DIC in the pre- and postcracking phase.

## 1. Introduction

Digital Image Correlation (DIC) is a noncontact measurement technique that is increasingly used in several engineering fields. DIC techniques provide full-field maps of response quantities like strains and displacements, which represent a more complete set of information than local measurements obtainable by traditional approaches. On the one hand, there are required (preliminary) operations of DIC such as the need to apply a stochastic pattern (sprayed pattern) to the surface of the material, which might not always be possible. On the other hand, there are many advantages of DIC, such as the relatively simple experimental setup, the adjustable spatial and temporal resolutions, the possibility of examining specimens with different dimensions and involved materials, and the adaptability to different loading rates ranging from quasistatic to dynamic [1]. What is particularly convenient in DIC is the way in which crack initiation and development throughout the specimen dimensions can be analyzed, a goal that cannot be accomplished via conventional (contact-based) measurement approaches, like displacement transducers and strain gauges. This occurs not only because strain gauges are capable to measure local values only but also because in most practical engineering problems the knowledge of the cracking pattern is unknown beforehand. Therefore, their installation location, generally based on engineering judgement, may turn out to be not really appropriate.

Some of the most interesting applications of DIC include the investigation of the mechanical behavior of concrete structures. Indeed, concrete is a heterogeneous material characterized by a complex fracture behavior, which is difficult to be analyzed with conventional contact measurement approaches like local strain gauges. Moreover, analyzing the failure process of concrete structures is challenging because cracks exhibit micrometric dimensions, are broadly diffused on the material, and rapidly evolve in the internal microstructure. In this regard, DIC proves to be a reliable full-field measurement approach, as demonstrated by the good results reported in previous studies. Choi et al. [2] were among the pioneers to use DIC to assess the nonuniform distribution of displacements and strains of concrete surfaces. Kozicki et al. [3] examined notched concrete beams tested under three-point bending and demonstrated the effectiveness of DIC to evaluate the strain field of the specimens and the crack propagation for different beam dimensions. Shah et al. [4] used DIC to study the mode I and II fracture toughness for a number of concrete-concrete joint interfaces tested under three-point bending. Wu et al. [5] employed DIC to study the fracture behavior of notched concrete beams, considering different beam dimensions and notch depths. The camera resolution is an important aspect for the reliability of the resulting DIC measurements. Alam et al. [6] applied DIC to monitor the crack initiation and propagation in beams having different dimensions. In the quoted experimental work, the crack growth was correctly identified but the elastic strains were not captured due to the low camera resolution. Recent applications of DIC include the quantification of shear cracks in reinforced concrete beams, as reported by Hu and Wu [7,8] and by Huang et al. [9].

Of particular relevance to this research work, DIC can be usefully employed to investigate the interface behavior between externally bonded composite systems and concrete substrate, which represents a widespread strengthening technique for retrofitting existing concrete structures. The oldest and most popular strategy consists of externally bonding fiber-reinforced polymer (FRP) [10] sheets or strips to concrete elements in order to increase the flexural and/or shear strength [11,12,13,14,15]. In this context, DIC techniques were found to be useful for the characterization of the bond behavior. Coor et al. [16] investigated the interfacial properties in a single-lap shear test in which a carbon FRP sheet was externally bonded to a concrete block; Mahal et al. [17] analyzed the fatigue behavior of reinforced concrete beams with externally bonded carbon FRP sheets and near-surface mounted bars. 

As a more recent alternative, fabric reinforced cementitious mortar (FRCM) sheets represent the natural evolution of FRP systems in which an inorganic (cement-based) matrix is used in place of an epoxy matrix [18,19]. The use of FRCM in place of FRP is motivated by a better compatibility with the concrete substrate, which reduces debonding phenomena especially under harsh environmental conditions [20,21], such as high temperatures (close to the glass transition temperature of the epoxy matrix in FRP) or high humidity conditions [22,23,24,25]. Experimental investigations demonstrated the effectiveness of externally bonded FRCM systems for different applications, such as flexural [26,27,28,29] and shear strengthening [30,31,32,33,34,35,36] as well as increase of the confinement action of reinforced concrete members [37,38,39,40,41]. A critical issue of FRCM systems concerns the interfacial bond behavior between FRCM and concrete, which may trigger different failure modes such as cohesive debonding of concrete substrate, fiber rupture, fiber sliding within the matrix, and detachment at matrix-to-substrate interface. The bond behavior at the FRCM-concrete interface was extensively studied in the literature, by means of analytical, numerical, and experimental approaches [42,43,44,45,46,47,48,49,50,51,52,53,54,55]. The strain transfer mechanism in polybenzoxole (PBO) FRCM-concrete interface was analyzed via embedded fiber Bragg grating sensors by Montanini et al. [56]. Similar to what was said above for FRP, DIC represents a useful monitoring technique to investigate the evolution of the strain distribution and to analyze the bond behavior at the FRCM-concrete interface. Sabau et al. [57] used DIC to investigate FRCM-concrete joints in single-lap shear tests and compared the DIC results to those obtained by traditional strain gauges. In a similar research context, D’Anna et al. [58] recently did the same for the tensile characterization of basalt FRCM composites.

Along such research line, in this paper an experimental application of DIC for the investigation of PBO FRCM-concrete interface bond behavior is documented. The novel contributions of this experimental work in comparison to other studies are summarized as follows:

- In the literature, FRCM-concrete bond behavior was generally investigated by single-lap shear tests wherein the load is directly applied to the strengthening system. Instead, in this paper the bond behavior is analyzed by an alternative notched beam test setup in accordance with ASTM D7958/D7958M standards [59]. In the proposed test setup, the load is indirectly transferred to the strengthening system due to bending deformation and, consequently, this loading scenario is more consistent with real configurations of flexural strengthening systems of concrete beams as observed in practical retrofitting cases.
- Experimental three-point bending tests on concrete notched beams with externally bonded PBO-FRCM sheets are conducted for specimens subject to different environmental conditions (humidity and temperature), in order to assess whether these factors influence the bond behavior [60].
- Mechanical tests are performed vis-à-vis DIC measurements using two distinct cameras simultaneously, one focused on the concrete front face and another focused on the FRCM-concrete interface. This experimental setup makes it possible to interpret the mechanical behavior and failure mode of the specimens not only from a traditional macroscopic viewpoint but also under a local perspective concerning the evolution of the strain distribution at the FRCM-concrete interface obtained by DIC.

## 2. Materials and Methods

### 2.1. Materials and Specimen Preparation

Prismatic concrete beams are prepared using an ordinary Portland cement. The characterization of the concrete properties is made in terms of compressive strength and flexural strength, evaluated on standard specimens at 28 days in accordance with UNI EN 12390-3:2009 and UNI EN 12390-5: 2002 standards, respectively. The mean compressive strength of six standard cubes of 150 mm side is 47 MPa, with a coefficient of variation (CoV) of 9%; the mean flexural strength of six prismatic specimens is 6 MPa, with a CoV of 1.92%. 

**Figure 1:**
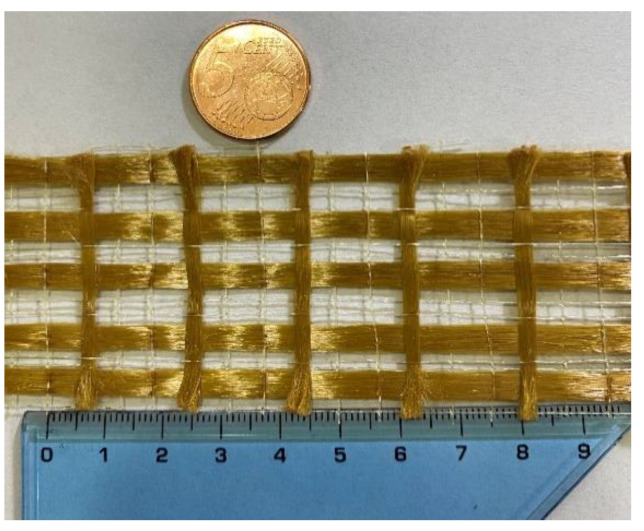
Structure of polybenzoxole (PBO) mesh used in the present research.

The FRCM is made of PBO mesh embedded in a cement-based mortar. The PBO bidirectional woven has an unbalanced configuration in the two directions, with fiber density higher in the main direction compared to the lateral direction, as shown in Figure 1. The properties of the PBO grid system determined in accordance with ASTM D3039 and ISO 527-5 standards are listed in Table 1. The mechanical properties of the cement-based mortar determined in accordance with UNI EN 196–1 standards are listed in Table 2.

**Figure 2:**
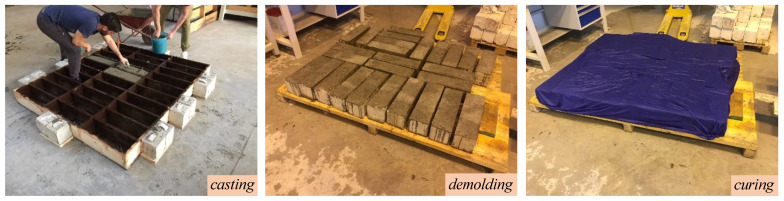
Preparation phases of concrete beams.

**Figure 3:**
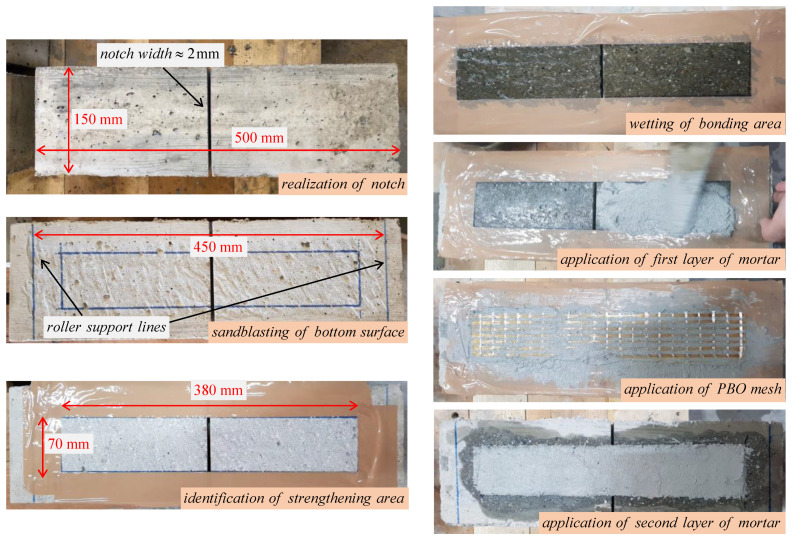
Notch realization and application of externally bonded PBO-FRCM sheet on the concrete beams.

**Figure 4:**
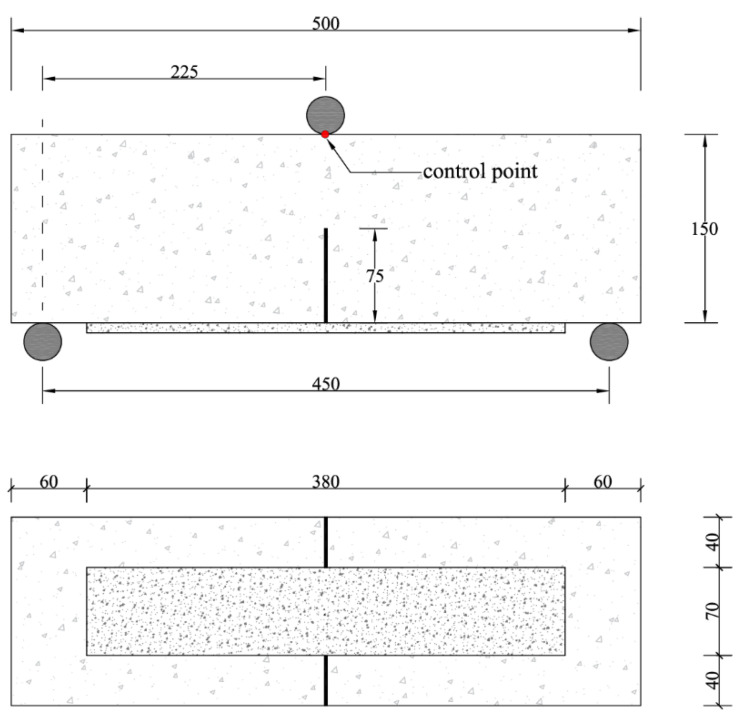
Final configuration of concrete beams with externally bonded PBO-FRCM sheet: front view (**top**) and bottom view (**bottom**). All sizes are reported in mm.

Prismatic concrete beams with nominal dimensions 150 × 150 × 500 mm are casted in preformed formworks, demolded after 24 h, and wrapped with a wet cloth to prevent water evaporation during the next 28 days, as shown in Figure 2. After 28 days, a band saw is employed to realize a notch starting from the mid-span of the specimen, with width equal to 2 mm and depth equal to 75 mm, in accordance with ASTM D7958/D7958M standards [59]. Then, the bottom surface is sandblasted and the strengthening area where to apply the FRCM sheet is identified. The application of the externally bonded PBO-FRCM sheet implies the following steps: (i) wetting of the strengthening area; (ii) application of the first layer of mortar (thickness 4 mm); (iii) application of the PBO mesh; (iv) application of the second layer of mortar (thickness 4 mm). The preparation phases of the notch realization and subsequent application of the externally bonded PBO-FRCM sheet on the bottom surface of the concrete beams are illustrated in Figure 3. The final concrete beam configuration with the externally bonded PBO-FRCM sheet for the three-point bending tests in accordance with ASTM D7958/D7958M standards [59] is reported in Figure 4.

### 2.2. DIC Fundamentals

DIC is a nondestructive and noncontact optical measurement technique widely used to investigate the distribution of strains and displacements of different structures [61]. This measurement approach is implemented in two stages: first, the target digital images are gathered, and then these images are processed through numerical correlation algorithms. Before the acquisition of the images, it is necessary to preliminarily prepare the specimen surface to be analyzed through a conditioning technique, by realizing an artificial stochastic pattern and/or point markers. This pattern is essential to track the evolution of the point position during loading, and to compute the corresponding mechanical quantities through techniques of DIC. The finer is the pattern mesh, the higher is the DIC quality. For these reasons, DIC requires the use of high-performance cameras to obtain accurate measurements [62].

**Figure 5:**
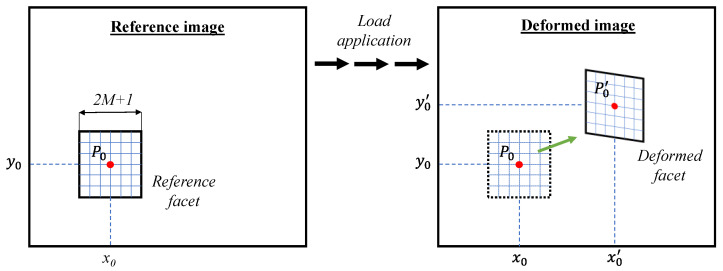
Schematic representation of the Digital Image Correlation (DIC) measuring process.

The fundamentals of the DIC measurement process [63] are illustrated in Figure 5. The reference image represents the initial condition of the specimen, which generally coincides with the unloaded configuration. This image is divided into subsets, termed facets, identified by an area of (2*M* + 1) × (2*M* + 1) pixels, where *M* is a positive integer number and other than 0. The subset size is critical to DIC accuracy, so it should be properly selected according to the actual random intensity distributions of the speckle patterns [64]. As the specimen is loaded, the facets are subjected to displacement and strain. Hence, the generic point *P*(*x*_0_; *y*_0_), centered in a reference facet, moves to the new position P’_0_(*x*’_0_; *y*’_0_) within the deformed facet. This displacement and the consequent strain are estimated using a cross-correlation (CC) or sum-squared difference (SSD) correlation algorithm, which identifies the similarity between the reference and the deformed facet. From a mathematical point of view, this involves searching for the peak of the distribution of the correlation coefficients. Specifically, the location of the target subset is identified, once the maximum or the minimum correlation coefficient has been found, depending on the used correlation criterion. Instead, the strain field can be obtained by numerical differentiation.

The registration and computation algorithms aim to ensure the best correlation between the interrogated facets. However, to achieve this goal, it is necessary to pursue a balanced compromise between spatial resolution, pattern speckle quality and computation time [65]. In fact, by reducing the size of the speckle pattern, it is possible to improve the accuracy of the DIC; however, the computation time increases considerably. Additionally, if the quality of the pattern speckle is poor, correlation procedures can introduce several errors and reduce the measurement accuracy.

### 2.3. Mechanical Test Setup

**Figure 6:**
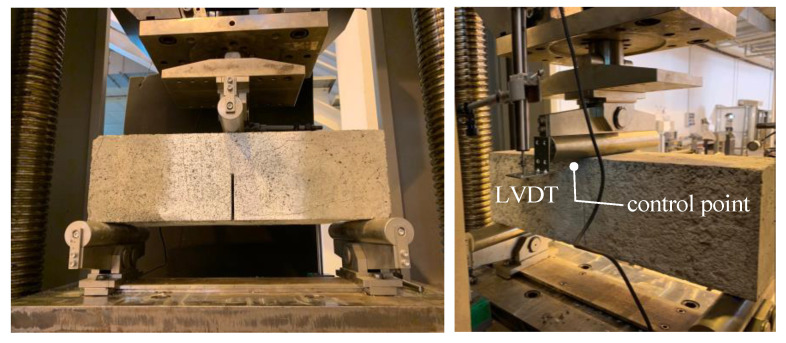
Notched concrete beams tested in three-point bending under displacement-controlled mode.

Three-point bending tests are performed according to ASTM D7958/D7958M standards [59], using a Zwick universal testing equipment with load capacity equal to 600 kN. The tests are conducted in displacement-controlled mode, with a constant displacement rate set as 0.05 mm/min. The displacement is monitored at a control point located at the top of the beam under the roller used for the load application, using a linear variable displacement transducer (LVDT) as illustrated in Figure 6. The load is monitored using a load cell integrated in the testing equipment. The tests are carried out until complete failure of the specimen, which is identified by a reduction of the load measured by the cell equal to 80%.

**Figure 7:**
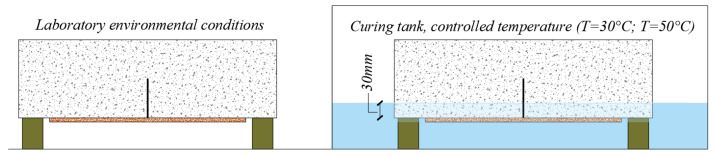
Curing conditions of the specimens considered in this experimental campaign.

The tests comprise different environmental conditioning of the specimens. After the application of the externally bonded PBO-FRCM sheet, three curing conditions are considered (see Figure 7):

- four specimens are cured in air at laboratory environmental conditions for 31 days (temperature 23 °C ± 2 °C and relative humidity 65–75%);
- four specimens are placed in a curing tank for 31 days, with their bottom face (depth equal to 30 mm) immersed in water (relative humidity 100%) at a controlled temperature of 30 °C;
- four specimens are placed in a curing tank for 31 days, with their bottom face (depth equal to 30 mm) immersed in water (relative humidity 100%) at a controlled temperature of 50 °C.

The goal of this investigation is to ascertain the influence of the environmental factors in the FRCM-concrete interface bond behavior. Apart from the laboratory conditions, two serviceability conditions are considered. The first conditioning temperature (30 °C) can be meant as a service temperature that is frequently experienced in summer seasons, while the second conditioning temperature (50 °C) can represent an ultimate limit state condition that is hardly exceeded in ordinary conditions.

### 2.4. DIC Test Setup

The use of correct lens and of high-resolution images plays a crucial role in the correct evaluation of the response and is essential for obtaining reliable results. For this reason, an appropriate DIC test setup has been conceived in this experimental campaign to ensure the highest resolution and a correct measurement [66,67].

**Figure 8:**
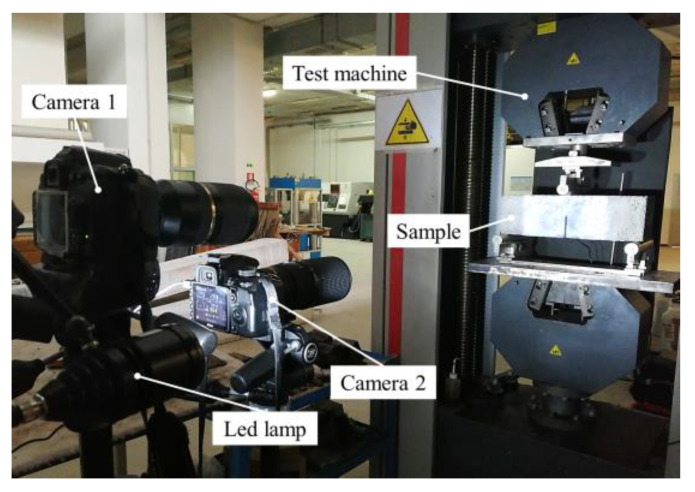
Experimental DIC test setup.

Two reflex cameras, models Nikon D3100 and Nikon D300s, are used in this work, called camera 1 and camera 2 in Figure 8 and in the sequel of the paper. These cameras are equipped with a macro lens (Nikon, AF Micro-Nikkor 200 mm f/4D IF-ED) and with a zoom lens (Tamron, SP AF 70-300 F/4-5.6 Di VC USD). The two distinct cameras are used simultaneously to investigate two regions of interest of the specimen: camera 1 is focused on the FRCM-concrete interface, while camera 2 is employed to examine the concrete front face, i.e., the area above the notch. In particular, camera 2 is useful to monitor the attainment of the tensile strength of concrete prior to the full engagement of the interface bond behavior (initial beam behavior). The position of the two cameras is determined after a series of trials, in order to ensure optimum field of view, focus and optical resolution. The final location of camera 1 and camera 2 are at a distance of 72 cm and 98 cm from the specimen surface, respectively. Two led lamps are used to guarantee a brightness of 1000 lux, constant throughout the test duration, in the overall region of the interest.

The synchronization of the acquisition is carried out by connecting a trigger port of the mechanical test equipment with a remote control for the two cameras. The rate of acquisition is set as one photo every 6 s, until the specimen failure. Depending on the lens models used in the two cameras, it is necessary to adopt different focal values, as listed in Table 3.

The set of digital images was elaborated using the DIC software GOM Correlate 2020 (GOM GmbH, Braunschweig, Germany).

## 3. Results and Discussion

### 3.1. Load-Deflection Curves

**Figure 9:**
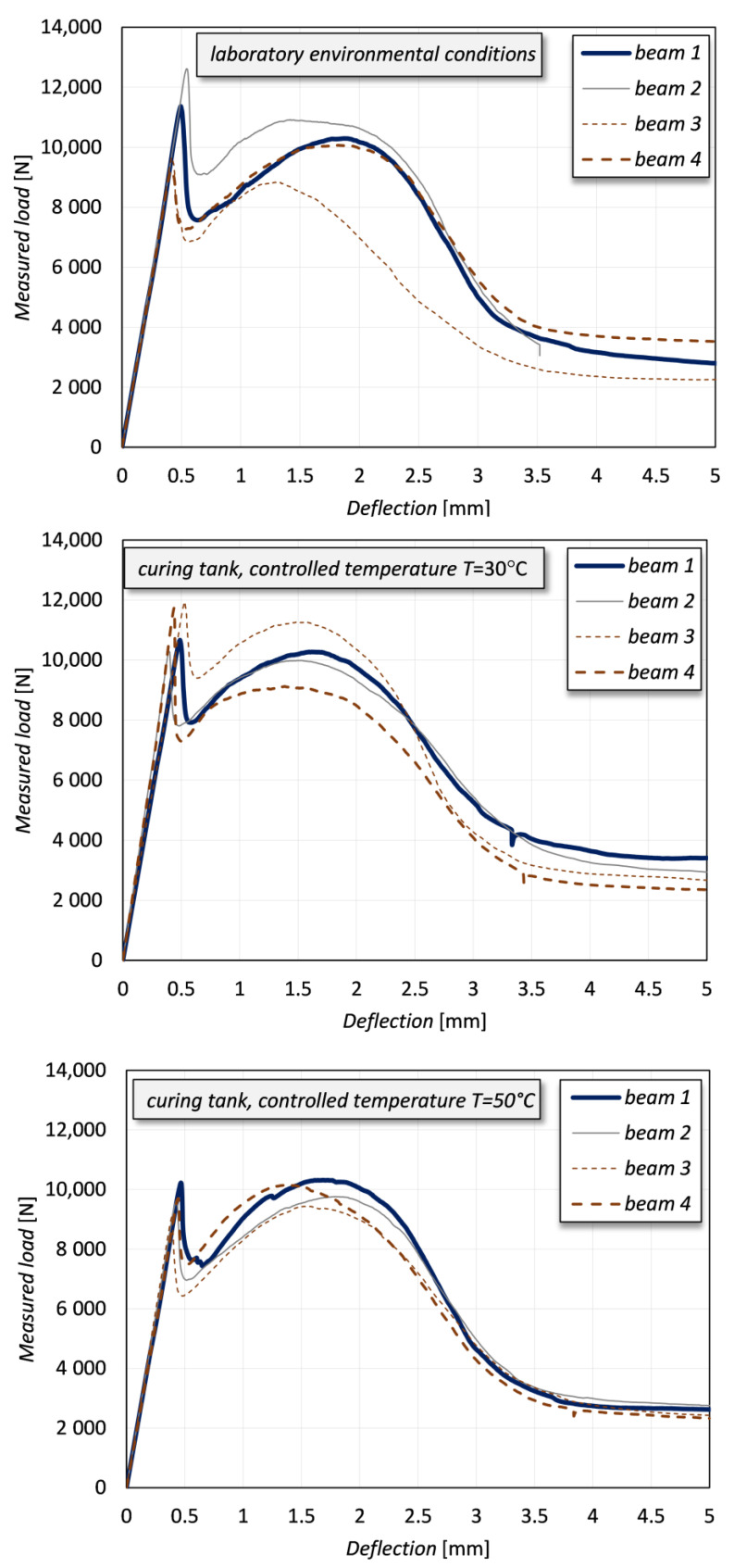
Results of the tests in terms of load-deflection curves for different curing conditions.

The results of the three-point bending tests on the notched concrete beams with externally bonded PBO-FRCM sheets are illustrated here in terms of load-deflection curves. The graphs in Figure 9 report the outcomes of the twelve specimens, namely four specimens for each curing condition separately. Although the observed behavior is comparable in all four specimens of each class, the slight variation of the obtained load-deflection curves is likely ascribed to the inherent uncertainty in the mechanical properties of the employed materials, especially concrete (CoV of the compressive strength equal to 9%). Interestingly, the discrepancies are more evident in the first case of laboratory environmental conditions, wherein the mechanical characteristics of concrete might have a more pronounced influence than in other curing conditions in which, instead, the overall behavior might be mainly governed by the interfacial bond properties at the FRCM-concrete interface. In general, the performance of the FRCM system is not negatively affected by temperature increase in the range considered in this experimental campaign. On the contrary, previous literature studies demonstrated the degradation of the bond behavior at the FRP-concrete interface under environmental conditions like high relative humidity and/or high temperature [22,23,24,25].

All the recorded curves show an initial elastic branch, in which the behavior of both concrete and FRCM composite system is linear. A clear peak is observed at a deflection equal to approximately 0.5 mm for all the specimens. Thereafter, a decrease of load occurs according to a typical trend of a concrete beam structure. The second part of all curves exhibits a local maximum and a slow nonlinear reduction of the measured load. This curve can be interpreted from a mechanical point of view, by also critically considering alternative single-lap shear tests available in the literature for the same material [68]. The peak at approximately 0.5 mm represents the attainment of the first cracking load of the beam, the subsequent decrease of load is due to the transfer of the system resistance to the FRCM composite and, finally, the shape of the curve is quite similar to the typical stress-global slip curve obtained by single-lap shear tests of FRCM-concrete interfaces [68].

**Figure 10:**
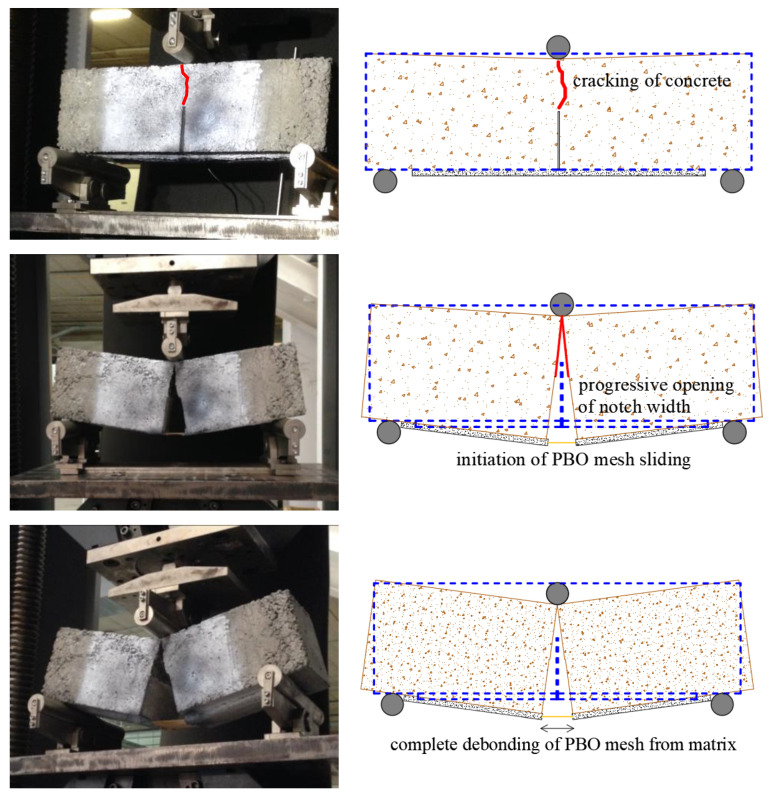
Results of the tests in terms of failure modes.

For all the beams, the complete failure is related to the PBO mesh debonding from the mortar matrix. As shown in Figure 10, the mortar matrix remains attached to the concrete substrate due to the good compatibility between the two cementitious materials. Upon progressive cracking of concrete region above the notch, an increase in the opening of the notch width is observed, until the beam is eventually split into two halves. The vertical deflection of the beam produces a rotation of the two halves of the beam with respect to the loading point under the roller support. In this condition, the load is entirely transferred to the FRCM fibers that, to accommodate the further increase of vertical deflection of the beam during the test, must detach progressively from the mortar matrix. Hence, a rigid body motion occurs up to complete PBO fiber sliding from the mortar matrix, cf. again Figure 10.

### 3.2. DIC Results

**Figure 11:**
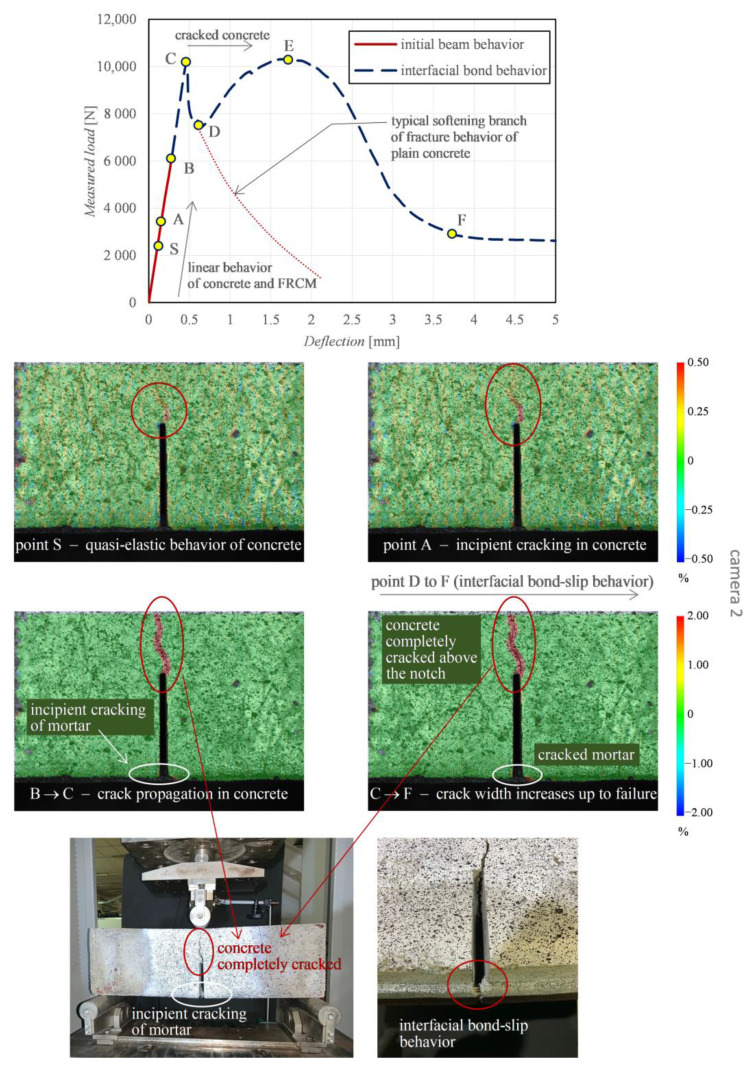
Representative beam: characteristic points in the load-deflection curve, and DIC-processed longitudinal strain maps from camera 2 examining the concrete front face.

**Figure 12:**
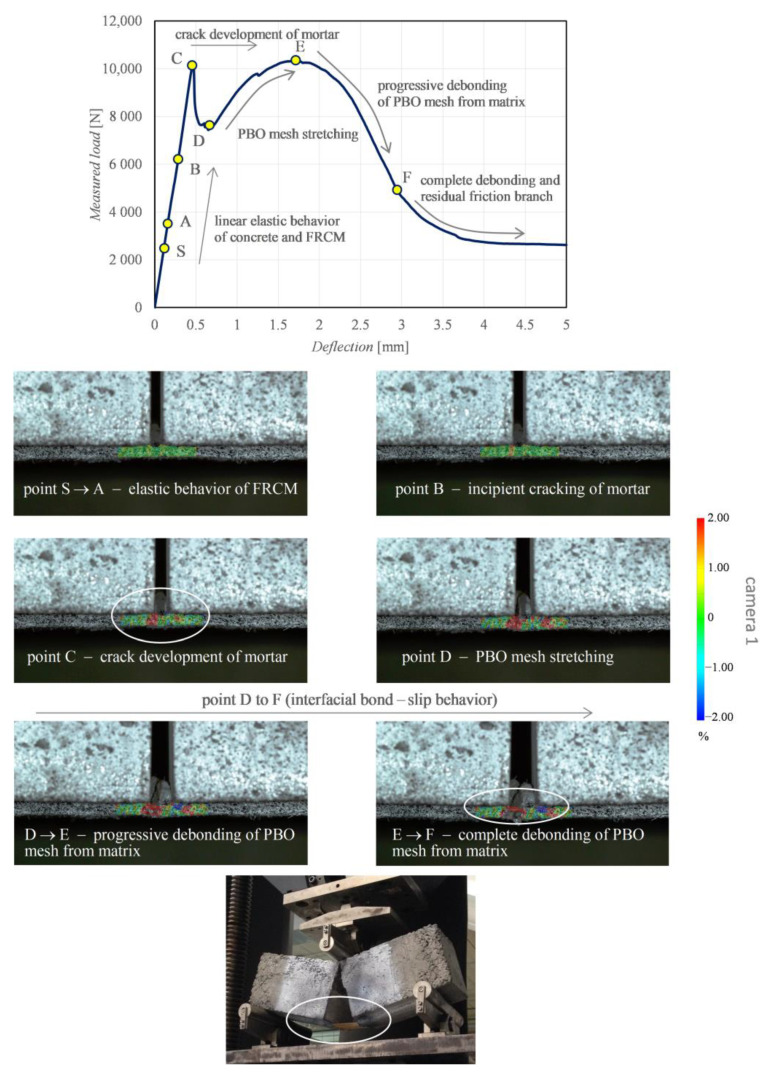
Representative beam: characteristic points in the load-deflection curve, and DIC-processed longitudinal strain maps from camera 1 examining the FRCM-concrete interface.

Considering the reasonably good agreement between the four repetitions of beams for each curing class, only some representative results among the tested specimens are selected and discussed concerning DIC test results. As said above, the two cameras allow the examination of two distinct regions of interest of the beams, namely concrete front face above the notch (camera 2) and FRCM-concrete interface (camera 1). In Figure 11 and Figure 12 the longitudinal strain results of a representative beam at controlled temperature of 50 °C are illustrated.

The DIC-processed images of the longitudinal strains refer to different stages of the loading application, which are identified by a set of characteristic points A-F in the relevant load-deflection curve (the point S represents a “starting point” close to the origin). The branch S-A denotes a linear elastic behavior of both concrete and FRCM. This is corroborated by the corresponding DIC strain distributions shown for the two regions of interest. Specifically, point A highlights the incipient cracking of concrete, while point B denotes the incipient cracking of mortar, with the concrete above the notch already partially cracked. By further increasing the load from point A to point B leads to the attainment of the tensile strength above the notch and, consequently, the neutral axis depth moves from the area close to the notch tip up to the loading point. This leads to the formation of a main crack above the notch, with a resulting tortuous pattern up to the top compressed fiber of the concrete beam (point B) and complete reduction of concrete contribution to the element strength, with the crack width gradually increasing (point C). The whole process is visible in terms of strain distribution between point A and point C obtained by camera 2 (Figure 11). Concurrently, the mortar matrix at the FRCM-concrete interface also exhibits some visible cracks that grow up further from point A to point C, which is confirmed by the strain distribution obtained by camera 1 (Figure 12). Precisely, at point B a strain intensification is observed, which corresponds to the incipient crack of a specific section of the FRCM mortar. At this stage, the resistance of the beam entirely relies on the FRCM contribution; hence, the load-deflection curve in Figure 11 can be separated in two branches, namely the initial beam behavior (from the origin up to point B) and the interfacial bond behavior (from point B up to failure).

In the initial beam behavior, the beam contribution and the FRCM contribution are mutually interacting. Once the concrete cracking and mortar cracking occur, the FRCM contribution prevails. In the interfacial bond behavior, the concrete is completely cracked, as shown in the DIC images. The load level C corresponds to the attainment of the mortar cracking. Once the critical deflection of point C (around 0.5 mm) is exceeded, a clear softening branch is observed from point C to point D, which is related to the residual tensile stress of concrete observed in the range of narrow crack widths [69,70]. This softening branch is similar to the behavior observed in fracture mechanics by testing a plain concrete prismatic beam specimen. In contrast to the case of a plain concrete specimen, in this case the beam is strengthened by the externally bonded FRCM sheet. Consequently, from point D the FRCM interacting contribution is involved since the PBO mesh stretches and, consequently, starts sliding from the mortar matrix, and a gradual increase of the load-carrying capacity is observed in the load-deflection curve. From point D the DIC images reveal that the sliding of the PBO grid from the mortar matrix becomes more manifest. The load first increases up to point E and then decreases from E to F. At point F, the load-deflection curve terminates with a typical horizontal friction branch, related to the residual strength at the FRCM-concrete interface [68].

**Figure 13:**
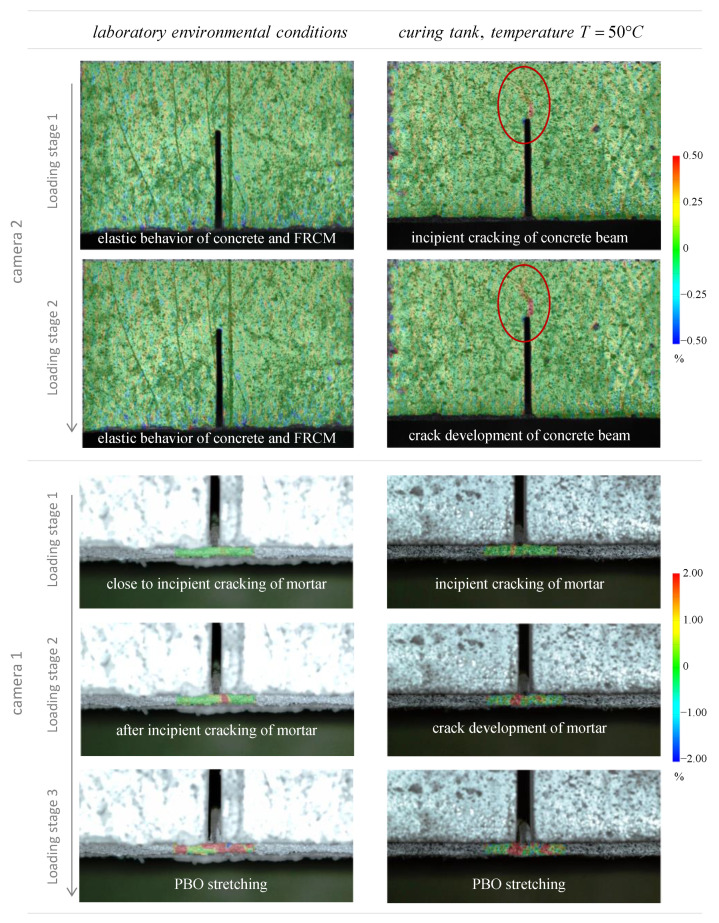
Comparison of DIC-processed images of longitudinal strains in the notched concrete beams under two different curing conditions.

It is worth noting that the characterization of the bond behavior at the FRCM-concrete interface is assisted by the DIC images, which allow a clearer interpretation of the mechanical behavior of the notched concrete beam, especially in the precracking phase. More specifically, the DIC-processed images explain the interaction of the two parallel resisting contributions in the specimens and the stress transfer mechanism with the increase of the vertical deflection of the beam. By inspection of the images related to the camera 1, it can be seen that the DIC strain maps are very useful to anticipate the incipient crack initiation of both concrete and mortar matrix under service conditions and the subsequent initiation and development of PBO mesh sliding from mortar matrix. To clarify this aspect and to investigate the effect of the different curing conditions of the samples, some DIC images aimed at capturing the occurrence of incipient crack and crack development are reported in Figure 13. Two representative specimens are compared, one cured at laboratory environmental conditions and one cured in tank at controlled temperature of 50 °C. Some loading stages (two for the concrete area above the notch and three for the FRCM-concrete interface, at equal loading level for the two curing conditions) are analyzed and compared. The images related to the camera 1 and 2 are reported at two different scales because the deformations corresponding to crack initiation are different in concrete and FRCM mortar. However, the same scale is adopted for the two curing conditions for comparison purposes. It is clearly seen that the curing condition slightly affects the mechanical behavior of the specimen in the cracking phase, something that is impossible to capture by the observation of the mere load-deflection curves. More specifically, for the specimens cured in tank at controlled temperature the concrete above the notch exhibits an incipient cracking at lower load levels compared to the specimens cured in laboratory environmental conditions. This seems to indicate that the temperature condition applied to the bottom part of the specimen has slightly altered the stress transfer mechanism at the FRCM-concrete interface, and, in turn, has generated a premature cracking of the concrete above the notch. On the contrary, after cracking of mortar takes place, the elongation of the PBO mesh increases until subsequent debonding from the mortar matrix, and this phenomenon is almost comparable in the two curing conditions, which is also confirmed by the qualitative agreement of the load-deflection curves observed for the two classes of specimens (see again Figure 9). Therefore, the macroscopic bond behavior is not significantly affected by the environmental conditioning.

**Figure 14:**
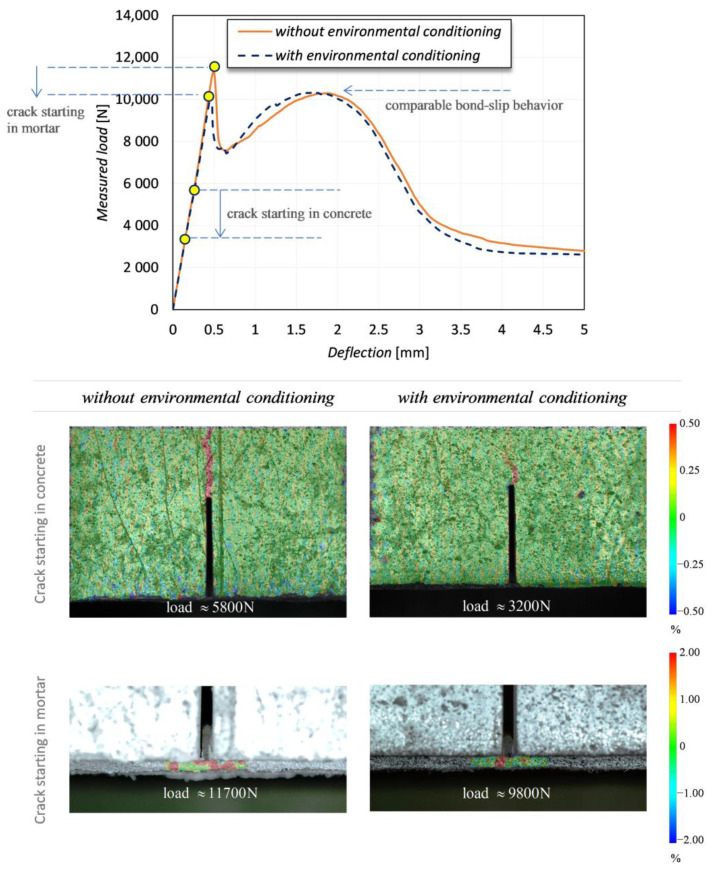
Crack starting in concrete and in FRCM mortar for specimens subject to different curing conditions.

Finally, Figure 14 focuses on the crack starting in concrete and in FRCM mortar. The load-deflection curves of the two analyzed specimens are shown in the top part of the figure, and the load levels corresponding to the crack starting of the two resisting elements (concrete and mortar) are also identified in the plot for the two curing conditions. Not only the concrete cracking but also the mortar cracking occurs at a lower load level in the specimen cured in tank at controlled temperature of 50 °C compared to the specimen cured in air at laboratory environmental conditions (i.e., without environmental conditioning). The crack load in concrete is almost halved in the specimen with environmental conditioning compared to the specimen without environmental conditioning, whereas the reduction of the crack load in mortar is less pronounced. The load-deflection curves exhibit an almost identical (qualitative and quantitative) behavior in the branches following the mortar cracking and representing the actual bond behavior at the FRCM-concrete interface (with the PBO mesh sliding from the underlying mortar matrix). This confirms that the curing conditions, in particular the effect of temperature and relatively humidity applied to the specimen, have a marginal influence on the interfacial properties of FRCM composite systems applied to concrete substrates, at least for the relatively short duration of the thermal treatment and temperature range involved in this experimental campaign. 

In conclusion, as demonstrated by these outcomes, the DIC technique can be used to assess the bond behavior at the FRCM-concrete interface or to determine the status of the interfacial properties after an exceptional event (e.g., seismic load), which could be useful to plan local repair interventions and, in general, for structural health monitoring purposes. 

**Figure 15:**
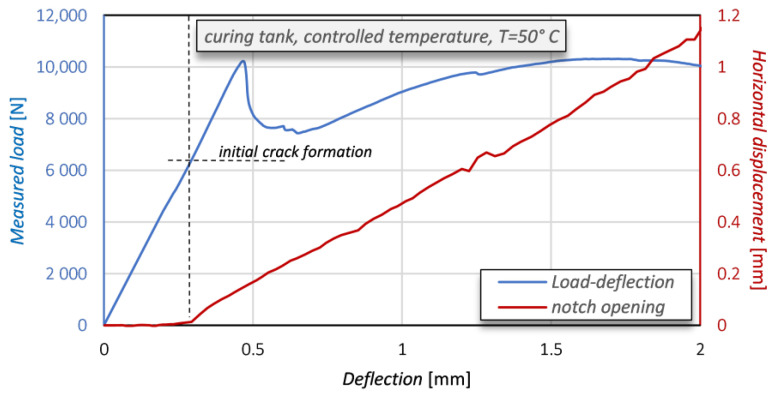
Opening of the notch of the concrete beam (in red) and load-deflection curve (in blue).

Figure 15 shows the opening of the notch in comparison with the load-deflection curve. The measurement of the notch opening has been obtained by monitoring the horizontal displacement of two points located on opposite faces of the notch, 1 cm below its upper boundary (notch tip).

After a first initial stage in which an almost zero displacement is observed, once the concrete beam has started its cracking then the notch opening grows linearly with the deflection. It is interesting to observe that the opening of the notch proceeds with an almost linear trend. Consequently, it is not significantly affected by the cracking of the mortar matrix and by the progressive debonding of PBO.

**Figure 16:**
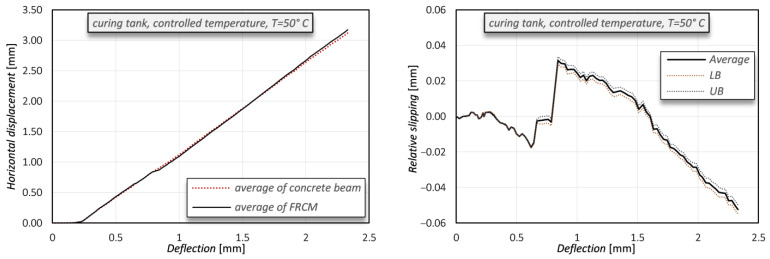
Comparison of average horizontal displacements measured on the concrete beam (black line) and on the FRCM (dotted red line) (**left**) and relative slipping between the concrete beam and the FRCM (UB = upper bound; LB = lower bound) (**right**).

Any slippage of the concrete beam with respect to the FRCM would highlight a limited adhesion capacity of the mortar matrix to the concrete substrate. Figure 16 (left) reports the average horizontal displacements measured on 10 corresponding points belonging to the concrete beam and to the FRCM, respectively, while Figure 16 (right) displays their difference (which can be meant as a “relative slipping”) together with upper and lower bounds (i.e., ± standard deviation). The maximum shift is below ±0.05 mm, thus proving that no practical relative slipping occurs between the beam and the reinforcement.

## 4. Conclusions

This paper has presented an experimental campaign focused on the characterization of the interfacial bond behavior between externally bonded PBO-FRCM sheets and concrete substrate, assisted by DIC. The FRCM-concrete interface bond behavior is investigated via DIC technique by using two cameras simultaneously, one focused on the concrete area above the notch and another focused on the FRCM-concrete interface. Three-point bending test on notched concrete beams in accordance with ASTM D7958/D7958M standards are carried out. Prior to testing, the specimens were subjected to different environmental conditions (humidity and temperature), in order to assess whether, and to what extent, these factors influence the bond behavior.

The main results of this research work are summarized as follows:

- Twelve specimens have been analyzed, and the qualitative trends of the load-deflection curves are quite similar and are marginally affected by the curing conditions. This demonstrates that the performance of the FRCM system is not negatively affected by temperature increase, at least for the relatively short duration of the thermal treatment and temperature range involved in this experimental campaign (partial immersion in water at 50 °C protracted for 31 days).
- After the attainment of cracking in concrete (above the notch) and in FRCM mortar (at the interface), the subsequent branches of the load-deflection curve are related to the stress transfer mechanism at the FRCM-concrete interface and resemble the typical stress-global slip curve observed in alternative single-lap shear tests available in the literature for the same material. For all the beams, the failure mode is ascribed to the PBO mesh debonding from the mortar matrix.
- DIC images of the longitudinal strains have facilitated the interpretation of the mechanical behavior of the specimens and have better explained the interaction of the two parallel resisting systems (concrete and FRCM composite), something that is impossible to capture by the observation of the mere load-deflection curves. These images have revealed that the temperature condition applied to the bottom part of the specimen has slightly altered the stress transfer mechanism with the increase of the vertical deflection of the beam in the precracking phase and has generated a premature cracking of concrete above the notch.
- By inspection of the DIC images it is clearly seen that the curing conditions affect the mechanical behavior of the specimen in the precracking phase only. On the contrary, after cracking of mortar takes place, the elongation of the PBO mesh increases until subsequent debonding from the mortar matrix, and this phenomenon is almost comparable in the two curing conditions, which is also confirmed by the qualitative agreement of the load-deflection curves observed for the two classes of specimens.

## Figures and Tables

**Table 1:** Mechanical and geometrical properties of PBO-fabric reinforced cementitious mortar (FRCM) composite system (fibers + matrix).

Parameter (Unit)	Value
Equivalent thickness (mm^2^/m)	0.046
Fabric width (mm)	70
Young modulus (GPa)	241
Tensile strength (MPa)	3421
Ultimate strain (%)	1.42
PBO fiber density (g/cm^3^)	1.56
PBO fiber tensile strength (MPa)	5800
PBO fiber ultimate strain (%)	2.5
PBO fiber Young modulus (GPa)	270

**Table 2:** Mechanical properties of mortar used in the PBO-FRCM composite system.

Parameter (Unit)	Value
Compressive strength at 28 days (MPa)	40
Flexural strength at 28 days (MPa)	4
Young modulus (GPa)	7

**Table 3:** Cameras optical parameters.

	F-Stop	Exposure	ISO Sensitivity
Macro lens	f/8	1/15 s	ISO—400
Zoom lens	f/8	1/10 s	ISO—250

## Data Availability

Data sharing not applicable.

## References

[1] Pan B. (2018). Digital image correlation for surface deformation measurement: Historical developments, recent advances and future goals. Meas. Sci. Technol..

[2] Choi S., Shah S.P. (1997). Measurement of deformations on concrete subjected to compression using image correlation. Exp. Mech..

[3] Kozicki J., Tejchman J. (2007). Experimental investigations of strain localization in concrete using Digital Image Correlation (DIC) technique. Arch. Hydro-Eng. Environ. Mech..

[4] Shah S.G., Kishen J.C. (2011). Fracture Properties of Concrete-Concrete Interfaces Using Digital Image Correlation. Exp. Mech..

[5] Wu Z., Rong H., Zheng J.-J., Xu F., Dong W. (2011). An experimental investigation on the FPZ properties in concrete using digital image correlation technique. Eng. Fract. Mech..

[6] Alam S., Loukili A., Grondin F. (2012). Monitoring size effect on crack opening in concrete by digital image correlation. Eur. J. Environ. Civ. Eng..

[7] Hu B., Wu Y.-F. (2017). Quantification of shear cracking in reinforced concrete beams. Eng. Struct..

[8] Wu Y.F., Hu B. (2017). Shear Strength Components in Reinforced Concrete Members. J. Struct. Eng..

[9] Huang Z., Tu Y., Meng S., Sabau C., Popescu C., Sas G. (2019). Experimental study on shear deformation of reinforced concrete beams using digital image correlation. Eng. Struct..

[10] National Research Council (CNR) (2013). Guide for the Design and Construction of Externally Bonded FRP Systems for Strengthening Existing Structures.

[11] Al-Rousan R., Issa M., Shabila H. (2012). Performance of reinforced concrete slabs strengthened with different types and configurations of CFRP. Compos. Part B.

[12] De Domenico D., Fuschi P., Pardo S., Pisano A.A. (2014). Strengthening of steel-reinforced concrete structural elements by externally bonded FRP sheets and evaluation of their load carrying capacity. Compos. Struct..

[13] Pisano A., Fuschi P., De Domenico D. (2013). Peak load prediction of multi-pin joints FRP laminates by limit analysis. Compos. Struct..

[14] De Domenico D. (2015). RC members strengthened with externally bonded FRP plates: A FE-based limit analysis approach. Compos. Part B Eng..

[15] Dong J., Wang Q., Guan Z. (2013). Structural behaviour of RC beams with external flexural and flexural-shear strengthening by FRP sheets. Compos. Part B Eng..

[16] Corr D., Accardi M., Graham-Brady L., Shah S. (2007). Digital image correlation analysis of interfacial debonding properties and fracture behavior in concrete. Eng. Fract. Mech..

[17] Mahal M., Blanksvärd T., Täljsten B., Sas G. (2015). Using digital image correlation to evaluate fatigue behavior of strengthened reinforced concrete beams. Eng. Struct..

[18] National Research Council (CNR) (2018). Istruzioni per la Progettazione, l’esecuzione ed il Controllo di Interventi di Consolidamento Statico Mediante l’utilizzo di Compositi Fibrorinforzati a Matrice Inorganica.

[19] ACI Committee 549 (2013). Guide to Design and Construction of Externally Bonded Fabric-Reinforced Cementitious Matrix (FRCM) Systems for Repair and Strengthening Concrete and Masonry Structures.

[20] Ghiassi B., Marcari G., Oliveira D.V., Lourenço P.B. (2013). Water degrading effects on the bond behavior in FRP-strengthened masonry. Compos. Part B Eng..

[21] Sciolti M.S., Frigione M., Aiello M.A. (2010). Wet lay-up manufactured FRPs for concrete and masonry repair: Influence of water on the properties of composites and on their epoxy components. J. Compos. Constr..

[22] Di Tommaso A., Neubauer U., Pantuso A., Rostasy F.S. (2001). Behavior of Adhesively Bonded Concrete-CFRP Joints at Low and High Temperatures. Mech. Compos. Mater..

[23] Ceroni F., Bonati A., Galimberti V., Occhiuzzi A. (2018). Effects of Environmental Conditioning on the Bond Behavior of FRP and FRCM Systems Applied to Concrete Elements. J. Eng. Mech..

[24] Ghiassi B., Lourenço P.B., Oliveira D.V. (2015). Accelerated Hygrothermal Aging of Bond in FRP–Masonry Systems. J. Compos. Constr..

[25] Toutanji H.A., Gómez W. (1997). Durability characteristics of concrete beams externally bonded with FRP composite sheets. Cem. Concr. Compos..

[26] Bencardino F., Carloni C., Condello A., Focacci F., Napoli A., Realfonzo R. (2018). Flexural behaviour of RC members strengthened with FRCM: State-of-the-art and predictive formulas. Compos. Part B Eng..

[27] Ebead U., Shrestha K.C., Afzal M.S., El Refai A., Nanni A. (2017). Effectiveness of Fabric-Reinforced Cementitious Matrix in Strengthening Reinforced Concrete Beams. J. Compos. Constr..

[28] Raoof S.M., Koutas L.N., Bournas D.A. (2017). Textile-reinforced mortar (TRM) versus fibre-reinforced polymers (FRP) in flexural strengthening of RC beams. Constr. Build. Mater..

[29] Raoof S.M., Bournas D.A. (2017). TRM versus FRP in flexural strengthening of RC beams: Behaviour at high temperatures. Constr. Build. Mater..

[30] Gonzalez-Libreros J.H., Sneed L.H., D’Antino T., Pellegrino C. (2017). Behavior of RC beams strengthened in shear with FRP and FRCM composites. Eng. Struct..

[31] Gonzalez-Libreros J.H., Sabau C., Sneed L.H., Pellegrino C., Sas G. (2017). State of research on shear strengthening of RC beams with FRCM composites. Constr. Build. Mater..

[32] Marcinczak D., Trapko T., Musiał M. (2019). Shear strengthening of reinforced concrete beams with PBO-FRCM composites with anchorage. Compos. Part B Eng..

[33] Loreto G., Babaeidarabad S., Leardini L., Nanni A. (2015). RC beams shear-strengthened with fab-ric-reinforced-cementitious-matrix (FRCM) composite. Int. J. Adv. Struct. Eng. IJASE.

[34] Tetta Z.C., Bournas D.A. (2016). TRM vs. FRP jacketing in shear strengthening of concrete members subjected to high temperatures. Compos. Part B Eng..

[35] Triantafillou T.C., Papanicolaou C.G. (2006). Shear strengthening of reinforced concrete members with textile reinforced mortar (TRM) jackets. Mater. Struct..

[36] Trapko T., Urbańska D., Kamiński M. (2015). Shear strengthening of reinforced concrete beams with PBO-FRCM composites. Compos. Part B Eng..

[37] Fossetti M., Alotta G., Basone F., Macaluso G. (2017). Simplified analytical models for compressed concrete columns confined by FRP and FRCM system. Mater. Struct..

[38] Gonzalez-Libreros J., Zanini M.A., Faleschini F., Pellegrino C. (2019). Confinement of low-strength concrete with fiber rein-forced cementitious matrix (FRCM) composites. Compos. Part B Eng..

[39] Faleschini F., Zanini M.A., Hofer L., Pellegrino C. (2020). Experimental behavior of reinforced concrete columns confined with carbon-FRCM composites. Constr. Build. Mater..

[40] Faleschini F., Zanini M.A., Hofer L., Toska K., De Domenico D., Pellegrino C. (2020). Confinement of reinforced concrete columns with glass fiber reinforced cementitious matrix jackets. Eng. Struct..

[41] Ombres L. (2014). Concrete confinement with a cement based high strength composite material. Compos. Struct..

[42] Raoof S.M., Bournas D.A. (2017). Bond between TRM versus FRP composites and concrete at high temperatures. Compos. Part B Eng..

[43] Raoof S.M., Koutas L.N., Bournas D.A. (2016). Bond between textile-reinforced mortar (TRM) and concrete substrates: Experimental investigation. Compos. Part B Eng..

[44] Carloni C., D’Antino T., Sneed L., Pellegrino C. (2015). Role of the Matrix Layers in the Stress-Transfer Mechanism of FRCM Composites Bonded to a Concrete Substrate. J. Eng. Mech..

[45] Carloni C., Santandrea M., Imohamed I.A.O. (2017). Determination of the interfacial properties of SRP strips bonded to concrete and comparison between single-lap and notched beam tests. Eng. Fract. Mech..

[46] Colombi P., D’Antino T. (2019). Analytical assessment of the stress-transfer mechanism in FRCM composites. Compos. Struct..

[47] D’Ambrisi A., Feo L., Focacci F. (2013). Experimental analysis on bond between PBO-FRCM strengthening materials and concrete. Compos. Part B Eng..

[48] D’Antino T., Carloni C., Sneed L.H., Pellegrino C. (2014). Matrix-fiber bond behavior in PBO FRCM composites: A fracture mechanics approach. Eng. Fract. Mech..

[49] D’Antino T., Sneed L.H., Carloni C., Pellegrino C. (2015). Influence of the substrate characteristics on the bond behavior of PBO FRCM-concrete joints. Constr. Build. Mater..

[50] D’Antino T., Sneed L.H., Carloni C., Pellegrino C. (2016). Effect of the inherent eccentricity in single-lap direct-shear tests of PBO FRCM-concrete joints. Compos. Struct..

[51] D’Antino T., Pellegrino C., Carloni C., Sneed L.H., Giacomin G. (2014). Experimental Analysis of the Bond Behavior of Glass, Carbon, and Steel FRCM Composites. Key Eng. Mater..

[52] Focacci F., D’Antino T., Carloni C., Sneed L., Pellegrino C. (2017). An indirect method to calibrate the interfacial cohesive material law for FRCM-concrete joints. Mater. Des..

[53] Ombres L. (2012). Debonding analysis of reinforced concrete beams strengthened with fibre reinforced cementitious mortar. Eng. Fract. Mech..

[54] D’Antino T., Focacci F., Sneed L.H., Carloni C. (2020). Relationship between the effective strain of PBO FRCM-strengthened RC beams and the debonding strain of direct shear tests. Eng. Struct..

[55] Sneed L.H., D’Antino T., Carloni C. (2014). Investigation of bond behavior of PBO fiber-reinforced cementitious matrix composite-concrete interface. ACI Mater J..

[56] Montanini R., Recupero A., De Domenico F., Freni F. (2016). Strain Sharing Assessment in Woven Fiber Reinforced Concrete Beams Using Fiber Bragg Grating Sensors. Sensors.

[57] Sabau C., Gonzalez-Libreros J., Sneed L.H., Sas G., Pellegrino C., Täljsten B. (2017). Use of image correlation system to study the bond behavior of FRCM-concrete joints. Mater. Struct..

[58] D’Anna J., Amato G., Chen J.F., Minafò G., La Mendola L. (2021). Experimental application of digital image correlation for the tensile characterization of basalt FRCM composites. Constr. Build. Mater..

[59] ASTM (2017). Standard Test Method for Evaluation of Performance for FRP Composite Bonded to Concrete Substrate Using Beam Test.

[60] De Domenico D., Urso S., Borsellino C., Spinella N., Recupero A. (2020). Bond behavior and ultimate capacity of notched concrete beams with externally-bonded FRP and PBO-FRCM systems under different environmental conditions. Constr. Build. Mater..

[61] Pan B., Li K. (2011). A fast digital image correlation method for deformation measurement. Opt. Lasers Eng..

[62] Montanini R., Rossi G., Quattrocchi A., Alizzio D., Capponi L., Marsili R., Di Giacomo A., Tocci T. Structural characterization of complex lattice parts by means of optical non-contact measurements. Proceedings of the 2020 IEEE International Instrumentation and Measurement Technology Conference (I2MTC).

[63] Pan B., Qian K., Xie H., Asundi A. (2009). Two-dimensional digital image correlation for in-plane displacement and strain measurement: A review. Meas. Sci. Technol..

[64] Montanini R., Squadrito G., Giacoppo G. (2011). Measurement of the clamping pressure distribution in polymer electrolyte fuel cells using piezoresistive sensor arrays and digital image correlation techniques. J. Power Sources.

[65] Bing P., Hui-Min X., Bo-Qin X., Fu-Long D. (2006). Performance of sub-pixel registration algorithms in digital image correlation. Meas. Sci. Technol..

[66] Allevi G., Castellini P., Chiariotti P., Docchio F., Marsili R., Montanini R., Pasinetti S., Quattrocchi A., Rossetti R., Rossi G. Qualification of additive manufactured trabecular structures using a multi-instrumental approach. Proceedings of the 2019 IEEE International Instrumentation and Measurement Technology Conference (I2MTC).

[67] Allevi G., Capponi L., Castellini P., Chiariotti P., Docchio F., Freni F., Marsili R., Martarelli M., Montanini R., Pasinetti S. (2020). Investigating Additive Manufactured Lattice Structures: A Multi-Instrument Approach. IEEE Trans. Instrum. Meas..

[68] Sneed L.H., D’Antino T., Carloni C., Pellegrino C. (2015). A comparison of the bond behavior of PBO-FRCM composites determined by double-lap and single-lap shear tests. Cem. Concr. Compos..

[69] ACI-ASCE Committee 445, ACI 445R–99 (1998). Recent approaches to shear design of structural concrete. J. Struct. Eng..

[70] De Domenico D., Ricciardi G. (2019). Shear strength of RC beams with stirrups using an improved Eurocode 2 truss model with two variable-inclination compression struts. Eng. Struct..

